# 5-HTP inhibits eosinophilia via intracellular endothelial 5-HTRs; SNPs in 5-HTRs associate with asthmatic lung function

**DOI:** 10.3389/falgy.2024.1385168

**Published:** 2024-05-23

**Authors:** Matthew T. Walker, Jeffrey C. Bloodworth, Timothy S. Kountz, Samantha L. McCarty, Jeremy E. Green, Ryan P. Ferrie, Jackson A. Campbell, Samantha H. Averill, Kenneth B. Beckman, Leslie C. Grammer, Celeste Eng, Pedro C. Avila, Harold J. Farber, William Rodriguez-Cintron, Jose R. Rodriguez-Santana, Denise Serebrisky, Shannon M. Thyne, Max A. Seibold, Esteban G. Burchard, Rajesh Kumar, Joan M. Cook-Mills

**Affiliations:** ^1^ Allergy-Immunology Division, Northwestern University Feinberg School of Medicine, Chicago, IL, United States; ^2^ Herman B Wells Center for Pediatric Research, Department of Pediatrics, Indiana University School of Medicine, Indianapolis, IN, United States; ^3^ Department of Microbiology and Immunology, Indiana University School of Medicine, Indianapolis, IN, United States; ^4^ University of Minnesota Genomics Center, Minneapolis, MN, United States; ^5^ Department of Medicine, University of California, San Francisco, San Francisco, CA, United States; ^6^ Department of Pediatrics, Section of Pulmonology, Baylor College of Medicine, Texas Children’s Hospital, Houston, TX, United States; ^7^ Veterans Caribbean Health Care System, San Juan, PR, United States; ^8^ Centro de Neumologia Pediatrica, CSP, San Juan, PR, United States; ^9^ Pediatric Pulmonary Division, Jacobi Medical Center, Bronx, NY, United States; ^10^ Department of Pediatrics, University of California, San Francisco, San Francisco, CA, United States; ^11^ Center for Genes, Environment, and Health and the Department of Pediatrics, National Jewish Health, Denver, CO, United States; ^12^ Division of Pulmonary Sciences and Critical Care Medicine, Department of Medicine, University of Colorado, Denver, CO, United States; ^13^ Division of Allergy and Clinical Immunology, Ann and Robert H Lurie Children’s Hospital of Chicago, Chicago, IL, United States

**Keywords:** 5-hydroxytryptophan, eosinophil, endothelial cell, serotonin receptors, FEV1

## Abstract

**Background:**

Previous research showed that 5-hydroxytryptophan (5HTP), a metabolic precursor of serotonin, reduces allergic lung inflammation by inhibiting eosinophil migration across endothelial monolayers.

**Objective:**

It is unknown if serotonin receptors are involved in mediating this 5HTP function or if serotonin receptor (HTR) single nucleotide polymorphisms (SNPs) associate with lung function in humans.

**Methods:**

Serotonin receptor subtypes were assessed by qPCR, western blot, confocal microscopy, pharmacological inhibitors and siRNA knockdown. HTR SNPs were assessed in two cohorts.

**Results:**

Pharmacological inhibition or siRNA knockdown of the serotonin receptors HTR1A or HTR1B in endothelial cells abrogated the inhibitory effects of 5HTP on eosinophil transendothelial migration. In contrast, eosinophil transendothelial migration was not inhibited by siRNA knockdown of HTR1A or HTR1B in eosinophils. Surprisingly, these HTRs were intracellular in endothelial cells and an extracellular supplementation with serotonin did not inhibit eosinophil transendothelial migration. This is consistent with the inability of serotonin to cross membranes, the lack of selective serotonin reuptake receptors on endothelial cells, and the studies showing minimal impact of selective serotonin reuptake inhibitors on asthma. To extend our HTR studies to humans with asthma, we examined the CHIRAH and GALA cohorts for HTR SNPs that affect HTR function or are associated with behavior disorders. A polygenic index of SNPs in HTRs was associated with lower lung function in asthmatics.

**Conclusions:**

Serotonin receptors mediate 5HTP inhibition of transendothelial migration and HTR SNPs associate with lower lung function. These results may serve to aid in design of novel interventions for allergic inflammation.

## Graphical Abstract

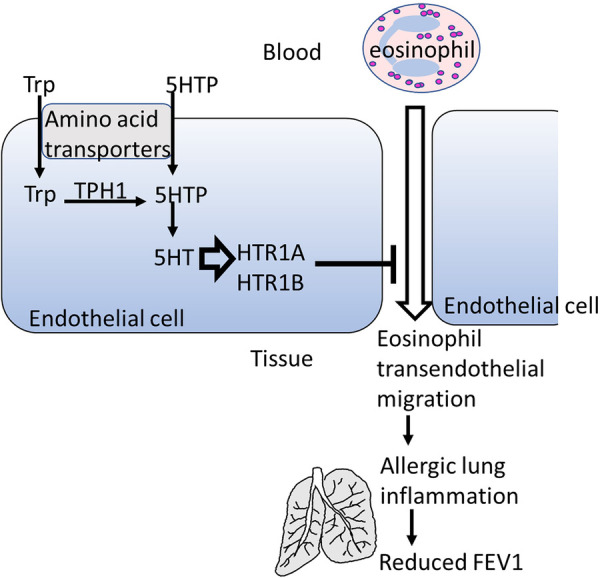

Model for 5-HTP regulation of eosinophil recruitment.

## 3 Key messages

• Serotonin receptors in endothelial cells are intracellular.
• Intracellular serotonin receptors in endothelial cells mediate 5-hydroxytryptophan inhibition of eosinophil transendothelial migration.
• In asthmatics, serotonin receptor SNPs associate with lower lung function.

## Introduction

Asthma affects up to 8% of adults and 7% of children (1). The most common subtype of asthma is allergic asthma, affecting up to 80% of children with asthma (2–5). A challenging aspect of asthma is that mental health disorders like anxiety and depression are more prevalent in people with asthma (6–11). A link between asthma and anxiety may lie within the serotonin pathway (12–19). Decreased serotonin synthesis or polymorphisms of the multiple classes of serotonin receptors (HTR's) are associated with anxiety and depression (20–29)*.* Furthermore, serotonin receptors are found on the surface of immune cells, endothelial cells, and immunoregulatory functions have been attributed to serotonin (15–19, 30, 31).

For serotonin synthesis, the rate limiting step is tryptophan hydroxylase (TPH) of metabolism of L-tryptophan to 5-hydroxytryptophan (5HTP). 5HTP is then rapidly metabolized to serotonin (Table 1A). Decreased TPH function reduces generation of 5HTP and serotonin, which is associated with depression (20–24). An imbalance in 5-HTP and serotonin levels can result in changes in activation of 14 subtypes of inhibitory and stimulatory HTR's in peripheral tissues (22, 61–64). Furthermore, single nucleotide polymorphisms (SNPs) in the *HTR*s alter HTR function or are associated with behavior disorders (21–23, 26, 27, 29, 65–71).

**Table 1:** Serotonin synthesis pathway and serotonin receptors.

A.				
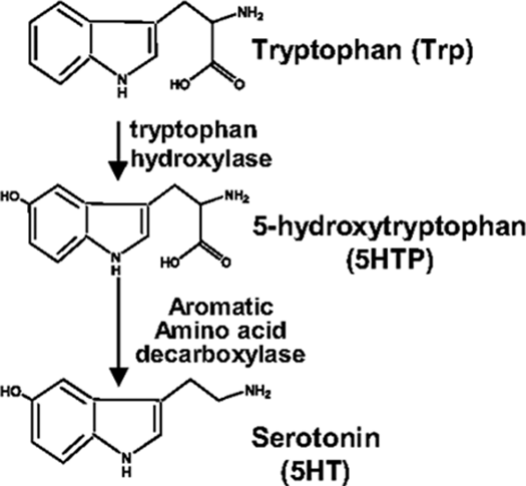				
B.				
Serotonin pathway genes	Chromosome locations	Chromosome markers for allergy	Allergy phenotype in reported associations	References
*TPH1*	11p15.1	D11S1981/D11S902-D11S899 (all are 11p15.1)	IgE and airway hyperresponsiveness	(32, 33)
		D11S1392 (11p13)	FEV1	(34)
		111p5.4 (*MRGPRE*), 11p15.5 (*MUC2*)	Asthma, FVC	(35)
*TPH2*	12q21.1	D12S355 (12q14.1), D12S1684 (12q21.2), D12S1667 (12q21.31), D12S81 (12q21.31), D12S351 (12q21.33), D12S95 (12q22), D12S327 (12q22)	HDM rast+.	(32)
		D12S351 (12q21.33)	Associated with asthma	(36)
		12q21.1	A peak for association with asthma	(37)
		D12S92 (12q21.1)	Association with atopy and allergy	(38)
		D12S83-D12S342 (12q24.3–q24.32)	Associated with asthma/IgE/eosinophils	(33, 39)
		12q23.3 (*CHST11*)	FEV1	(40)
*HTR1A*	5q12.2	5q11.2–q14.3	Associated with serum IgE	(41)
			HTR1A near peak for FEV1 on 5q but broad curves shown	(34)
		D5S647 (5q12.3), *PDE4D*	Associated with asthma	(42, 43)
*HTR1B*	6q14.1	6q13	Associates with eosinophils	(36)
		6q15	Asthma	(39, 44)
*HTR1D*	1p36.12	D1S478 (1p36.12), D1S234 (1p36.11), *MAFP2*	Associated with IgE, FEV1/FVC	(32, 35, 43)
		1p36.21 (*DHRS3*)	Asthma, FVC	(35, 45)
		1p36.31 (*ACOT7*)	IgE	(46)
		1p36.33 (*SKI*)	FEV1	(40)
*HTR2A*	13q14.2	13q13.2	Asthma	(47)
		*HTR2A* is at D13S153 (13q14.2)	Association with atopy and asthma	(38)
		*HTR2A* is within D13S1297-D13S796 (13q14.11–13q33.3)	Association with atopy/RAST/asthma/eosinophils	(33)
		D13S328 (13q14.13–q14.2)	Atopy	(48)
		13q14	Skin test for allergy, FEV1, IgE locus	(43, 49, 50)
*HTR2C*	xq23	right adjacent to *IL-13RA* gene in the Xq23	13RA functions in both IL13 and IL4 responses.	(51)
*HTR3A HTR3B*	11q23.2	11q22.2 (*BIRC3*)	FEV1, FVC	(40)
		D11S1986 (11q23.1) to D11S1998 (11q23.3)	Atopy in African-Americans and Caucasians	(43, 52)
		D11S4464 (11q24.1)	FEV1	(34)
		D11S968 (11q25)	Eosinophilia in asthma, IgE	(32, 36)
*HTR4*	5q33.1	5q31.1 (*IL13, SEPT8, SOWAHA, NMUR2*), 5q31.3 (*GNPDA1, NDFIP1*)	Asthma	(44, 53, 54)
		*HTR4* is just to the right of *SPINK5,6,7,9*	In allergy/atopy QTL	www.genome.ucsc.edu
		5q31–33, 5q31.1–q31.2 and 5q32 and 5q33.3	Lung function, FEV1, asthma, IKZF2,HTR4, RAD50, ADRA1B, nearby IL13, IL4, SPINK5	(34, 40, 43, 55, 56)
		5q33.1	Mite-sensitive asthma	(57)
*HTR5B*	2q14.1	2q12.1, *IL1RL1*	Asthma, eosinophilia	(43, 58)
		2q13	Asthma	(45)
		2q14,	Asthma	(59)
*HTR7*	10q23.31	10q22	FEV1	(43)
		10q23.2 (*WAPL*), 10q23.31 (*PANK1*), 10q24 (*PDCD4*)	IgE, asthma	(43, 46, 60)

(**A**) Serotonin synthesis pathway. (**B**) Chromosomal locations of genes of the 5HTP/serotonin pathway that are near or within chromosome locations associated with allergic/asthmatic disease. Listed are the chromosomal locations for 5HTP/serotonin pathway genes that have functional polymorphisms in anxiety/depression. Then, in the left two columns, is aligned the chromosome markers that have been consistently reported as associated with asthma/allergy phenotypes. We found there is considerable overlap for this limited number of chromosomal locations. The locations for *HTR5A* (7q36.2), and *HTR6* (1p36.13) were not in chromosome locations associated with Allergy Phenotype from GWAS studies.

HTRs and serotonin synthesis also exist in peripheral tissues. Interestingly, systemic administration of serotonin and 5HTP have opposing effects in the vascular and immune systems. Systemic administration of serotonin induces smooth muscle constriction in the airway (72–74). Also, vasoconstriction of the pulmonary arteries and aorta (75–77) can occur during treatment of anxiety and depression with selective serotonin reuptake inhibitors (SSRI's), which block the neuronal SERT transporter and elevate systemic serotonin levels (78). On the other hand, 5HTP is associated with vasodilation in animal studies (79–82). The opposing functions of 5HTP and serotonin on vasoconstriction may occur as an outcome of changes in local microenvironment concentrations of 5HTP and serotonin that would generate a change in the balance of activation of the multiple inhibitory and stimulatory HTRs that are differentially expressed by cells. Pharmacological inhibitors of the individual serotonin receptors HTR1A and HTR2A had only marginal benefit for asthma as summarized in a review by Cazzola et al. (83). It is anticipated that because there are multiple HTR receptors, a balance of multiple HTRs functioning in concert may be needed to regulate asthma.

It has been shown that 5HTP is acquired by endothelial cells and is metabolized to serotonin within the endothelial cells and that 5HTP reduces leukocyte transendothelial migration and reduces eosinophilic inflammation in four preclinical models and reduces airway hyper-responsiveness (18). Although 5HTP supplementation blocked leukocyte recruitment in the preclinical models, it did not alter leukocyte and eosinophil adhesion molecule expression, chemokine receptor expression, chemotaxis, or chemokinesis (18). However, whether HTRs have a role in 5HTP regulation of endothelial cells and leukocytes in allergic inflammation is not known. Moreover, these preclinical studies used the natural product 5HTP, derived from the plant *Griffonia simplifonia*, suggesting that it may act as an innovative novel therapy to address the current treatment gaps for allergic asthma and mood disorder.

We report subtypes of HTRs for mouse endothelium and eosinophils. Furthermore, selective HTR1A and HTR1B pharmacological inhibitors or siRNA knock down of these receptors blocked 5HTP inhibition of migration without altering eosinophil adhesion. In contrast, inhibition or siRNA knock down of these receptors in eosinophils did not block the 5HTP inhibition of transendothelial migration or eosinophil binding. Thus, endothelial serotonin receptors rather than eosinophil serotonin receptors, mediated 5HTP inhibition of transendothelial migration. Furthermore, 5HTP was catabolized to serotonin in endothelial cells and, intriguingly, HTR expression was intracellular in the endothelial cells. Consistent with intracellular HTR expression in endothelial cells and the inability of serotonin to cross cell membranes, exogenous addition of serotonin did not inhibit eosinophil transendothelial migration. In the CHIRAH and GALA cohorts, indexes of SNPs in *HTR*s and *TPH* associated with decreased lung function. These results will help design clinical studies addressing mechanisms for 5HTP inhibition of allergic asthmatic responses and the common comorbidities of anxiety or depression.

## Methods

### Animals

C57BL/6J mice (female, 6–8 weeks old) and NJ1638 mice were from Jackson Laboratory, Bar Harbor, Maine. The studies are approved by the Indiana University Institutional Review Committee for animals (protocol number: 21109).

### CHIRAH and GALA cohorts

#### CHIRAH

This analysis is based on the cohort established by the Chicago Initiative to Raise Asthma Health Equity (CHIRAH) study (84–86). The institutional review board of Northwestern University approved the CHIRAH protocol (IRB Project Number: STU00042117) (84–86). Written informed consent was obtained. The CHIRAH cohort is a community-based longitudinal cohort study of urban children and adults with persistent asthma enrolled from February 2004 to July 2005. The cohort was established by a broad community-based screening for households with persons with asthma using a school-based sampling technique. Adults were analyzed in this study. The adults were 18–40 years of age and self-reported race/ethnicity as non-Hispanic/African American, Hispanic/Latino, and non-Hispanic white/other. Our analyses examined the adults that had lung function analyses and DNA samples and were self-identified as African Americans (*n* = 103) or Latinx (*n* = 73) (85, 87, 88). Key SNPs in the *HTR1A/1B/2A/2C/3B* and *TPH1* and *TPH2* were chosen by effects on gene function or associations with behavior disorders (21–23, 26, 27, 29, 65–71). SNP analyses were carried out using the sequenom iPLEX platform.

#### GALA II

The Genes-environments and Admixture in Latino Americans (GALA II) is a clinic-based multicenter asthma case-control study designed to examine the genetic and environmental risk factors for asthma and asthma-related phenotypes in Latino children (Institutional Review Board of University of California San Francisco, IRB project number 10-00889; Ann and Robert H Lurie Children's Hospital of Chicago, IRB project number 2008-13531) (89–91). Written informed consent was obtained. Asthma cases were defined as participants aged 8–21 years with a history of physician-diagnosed asthma and the presence of 2 or more symptoms of coughing, wheezing, or shortness of breath in the 2 years preceding enrollment. Healthy control subjects were recruited from the community and clinics with the same catchment area as cases. Subjects were recruited from five centers (Chicago, Illinois; Bronx, New York; Houston, Texas; the San Francisco Bay Area, California; and Puerto Rico) from July 2008 through November 2011. Control subjects were frequency matched on age (within 1 year), sex, and study center. 4,477 subject participants were considered for inclusion in the study sample. After excluding those with missing values for key variables, the final sample included 2,126 participants (90). For the GALA 2 analysis, genotyping had been carried out by the Affymetrix Axiom World Array 4™ (92). GALA protocols were approved by institutional review boards for the centers as previously described (89–91).

### Endothelial cells and eosinophils

Human microvascular endothelial cells from the lung (HMVEC-Ls) (CC-Lonza, Walkersville, MD) were grown in EGM-MV endothelial growth medium plus 5% FCS (catalog #CC-3125, Lonza) and were used at passage 2–6. To prepare human eosinophils, human peripheral blood was diluted 1/2.5 with PBS containing 2 mM EDTA and then separated on 1.090 g/ml Percoll with centrifugation at 1,200 rpm for 20 min at room temperature. The layer containing eosinophils and neutrophils was collected and red blood cells removed by hypotonic lysis. The cells were further isolated by depletion of neutrophils using human CD16 negative selection using Miltenyi microbeads (catalog #130-045-701) to yield >95% eosinophils as determined by morphology and eosin staining of cytospins. These cells were used for western blot analyses of serotonin receptor expression.

The murine endothelial cell line mHEVa was cultured as previously described (93, 94). The murine endothelial cells were maintained in RPMI 1,640 (Corning 15-040-CV) supplemented with 20% fetal calf serum, 2 mM L-glutamine (Corning), 1 mM HEPES (Corning), 10 mM Sodium bicarbonate (Sigma), 50 µg/ml gentamycin (Lonza), 1X Penicillin/Streptomycin (Corning). Spleen eosinophils from NJ1638 mice were used for transendothelial migration assays. To generate murine eosinophils for knockdown of eosinophil HTRs, bone marrow cells were flushed from tibia and femur of C57Bl/6 mice and cultured in RPMI 1,640 supplemented with 20% fetal calf serum, 2 mM L-glutamine, 2.5 mM HEPES, 50 µg/ml gentamycin, 1X Penicillin/Streptomycin (Corning), 0.1 mM Sodium Pyruvate (Corning), 5 µM beta-mercaptoethanol (Sigma), 100 ng/ml SCF (250-03-100UG), and 100 ng/ml FLT3-L (Peprotech 250-31l-100UG). After five days of bone marrow in culture the cells were treated with siRNAs (1 µM) or untreated, bone marrow cells are centrifuged and replaced with fresh media containing 10 ng/ml IL5 (Peprotech 215-15-50UG). On day 8, the cells were >85% eosinophils as determined by morphology and eosin staining of cytospins.

### Immunolabeling HTRs in cells for confocal microscopy

Cultured mouse endothelial cell monolayers were fixed in −20°C methanol for 15 min and rehydrated with PBS for 1 h, which removes free serotonin. The samples were blocked with goat serum in PBS-0.3% BSA, incubated with rabbit anti-mouse HTR1A (catalog #MBS2528551, MyBiosource), rabbit anti-mouse HTR1B (catalog #NB100-56350, Novus Biologicals), or isotype control antibodies for 1 h at room temperature, washed, labeled with FITC-conjugated goat anti-rabbit IgG antibodies (catalog #554020, BD Biosciences), washed and then labeled with Alexa Fluor® 532-conjugated rat anti-mouse VCAM-1 (catalog #NBP2-50620AF53, Novus Biologicals), washed and cover slipped with ProLong™ Gold Antifade Mountant with DAPI (catalog #P36935, ThermoFisher). Images were analyzed by fluorescence microscopy.

### Transfection with siRNA

The cell/RNA mixture was added to an electroporation cuvette (catalog #12358-346, Bulldog-bio) and pulsed twice at 125 V for 2.5 ms at a 50.0 ms interval followed by five pulses at 20 V for 50 ms at a 50.0 ms interval in a Nepa Gene electroporator. Cells were immediately placed in fresh media in a parallel plate flow chamber for transendothelial migration assays or transferred to cell culture flask for growth prior to western blot analysis. After 72 h in culture, cells were assayed for transendothelial cell migration or for HTR expression by western blot analysis.

### Western blots

For western blot analysis of siRNA knockdown of HTRs at 72 h after siRNA transfection, the cells were suspended with 0.03% EDTA, centrifuged and lysed in 1X RIPA buffer (Thermo 89900), on ice for 30 min. Protein concentration for each sample was determined via BCA assay (ThermoFisher 23227) and normalized. Loading buffer was added and 10 µg total protein was loaded onto 4%–20% Tris-Glycine gels (ThermoFisher XP04200) for western blot analysis of HTR1A expression or 40 µg total protein was loaded for western blot analysis of HTR1B expression. Blots were transferred to PVDF and blocked with 5% bovine serum albumin (Sigma) or 5% milk (BioRad) in TBS (Fisher) with 0.1% Tween20 (Fisher). Primary antibodies included rabbit anti-HTR1A (catalog #MBS2528551, MyBiosource) at 1:1,000, rabbit anti-HTR1B (catalog #NB100-56350, Novus Biologics) at 1:1,000, and rabbit anti-beta actin (catalog #12620, Cell Signaling) at 1:10,000. The secondary antibody was alpaca anti-rabbit conjugated to HRP (catalog #611-035-251, Jackson ImmunoResearch) at 1:10,000. Chemiluminescence was performed using WesternBright ECL substrate (Advansta R-03027-C50).

To examine endothelial cell surface HTR1A and HTR1B, serum-containing medium was removed from the endothelial cell monolayers by washing 3 times with phosphate buffered saline supplemented with 0.2 mM CaCl_2_ and 0.1 mM MgCl_2_ (PBS-Ca-Mg) because cations are required for cell adhesion. Then the surface of endothelial cells was biotinylated with 0.5 mg/ml sulfo-NHS-biotin (catalog #A39256, Fisher Scientific) in PBS-Ca-Mg for 30 min (95). Then, the cells were washed 3 times with 4°C 10 mM glycine in PBS-Ca-Mg to quench unbound sulfo-NHS-biotin and then wash once with 4°C PBS-Ca-Mg. The cells were collected by gentle scraping, centrifuged 1,200 rpm for 8 min, and lysed with a lysis buffer (150 mM NaCl, 20 mM Tris-HCl, 0.5% NP40, 10% glycerol, pH7.5 with protease phosphatase inhibitors) as described (95). The samples were vortexed briefly and incubated 10 min; this was repeated for a total of 3 times. The samples were centrifuged 13,000 rpm for 30 min at 4°C. The supernatant was collected and placed in a pre-chilled tube and then 100 µl of Pierce High-Capacity Streptavidin Agarose (catalog #20357, Thermo Scientific) was added and end-over-end rotation was applied for 2 h at 4°C. The samples were centrifuged 10,000 × g for 2 min at 4°C, supernatants were removed, and agarose beads were washed twice with lysis buffer, twice with 1.5 M guanidine HCl, and then twice with lysis buffer. SDS loading buffer was added to the samples, boiled for 5 min and centrifuged 5 min 10,500 × g for 2 min at 4°C. Samples were examined by western blot for HTR1A, HTR1B, VCAM-1 and βactin as described above.

### *In vitro* eosinophil migration assays in transwells or under laminar flow

For Transwell migration assays, confluent monolayers of mouse endothelial cells grown in slide flasks were treated with siRNA (HTR1A, HTR1B or scrambled) and grown for 3 days to confluence in slide flasks or confluent monolayers of endothelial were treated overnight with or without small molecule pharmacological inhibitors of HTR1A (6 nM NAD-299 and 150 nM WAY-100135), HTR1B (500 nM NAS-181 and 88 nM SB224289) or HTR3 (75 nM Dolasetron Mesylate and 62 nM Ondansetron Hydrochloride) (Tocris Bioscience, MN). These inhibitors are lipid soluble (datasheets, Tocris Bioscience, MN), and have been described to cross tissue membranes (96, 97). The treatments had no effect on cell viability as determined by trypan blue exclusion. At 18 h after treatment, eosinophil binding to endothelial cells and eosinophil transendothelial migration were assessed as we previously describe in detail (98, 99). For eosinophil binding, mouse eosinophils (>85% eosinophils) were added to a endothelial cell monolayers in a parallel plate flow changed and incubated at 37°C for 5 min; then nonbound eosinophils were removed by washing 3 times with PBS with 200 µM MgCl_2_ and 150 µM CaCl_2_. The bound cells were fixed with methanol, coverslipped and counted by microscopy. Eosinophil transendothelial migration was examined using a parallel plate flow chamber under conditions of laminar flow of 2 dynes/cm^2^, as previously described (98, 99). This migration assay is dependent on endothelial cell expression of VCAM-1 and the chemokine MCP-1 (93, 100). Eosinophils were added to the endothelial cell monolayers, laminar flow applied for 15 min, cells fixed with methanol, slides coverslipped and then cells that had migrated under the endothelial monolayer examined by phase contrast microscopy as we previously described (98, 99).

### Statistics

For the preclinical studies, data were analyzed by a one-way ANOVA followed by Tukey's or Dunnett's multiple comparisons test (SigmaStat, Jandel Scientific, San Ramon, CA). *p* < 0.05 is considered significant. Presented are the means ± the standard errors.

For analyses of the GALA2 and CHIRAH cohorts, the minor variants of the identified SNPs were evaluated for direction of association with percent predicted FEV1 as a positive or inverse association. To evaluate significance of the association of individual SNPs with lung function, linear regression was carried out in each cohort with FEV1 as the outcome. Covariates for the CHIRAH analyses included duration of asthma, household income, private insurance, household smoke exposure, and use of an inhaled corticosteroid. Covariates included for the GALA2 study are as follows: ancestry (African and European), whether the subject ever smoked tobacco, site, annual household income, and maternal education. The outcome for these analyses in both cohorts was percent predicted FEV1 using Hankinson NHANES equations (101). For the GALA2 and CHIRAH cohorts, the polygenic index is the sum of SNPs whereby coding for each SNP was 0, 1, or 2 for number of alleles with the SNP. SNPs included in the index in CHIRAH had a nominal association with FEV1 (*p* < 0.2). This *p*-value was chosen to allow for inclusion of SNPs which would not have met significance at the 0.2 level, to allow for inclusion on the basis of putative biological functional because these SNPs affect protein function or are associated with behavior disorders (21–23, 26, 27, 29, 65–71). For the larger cohort GALA, we included all SNPs with *p*-values <0.2. Then, using the allele coding for these SNPs in *HTR*s and *TPH*, a polygenic risk score was calculated for subjects in CHIRAH and GALA. In both cohorts, we carried out linear regression analyses of the polygenic risk scores on the outcome of percent predicted FEV1.

## Results

### HTRs are in chromosome locations associated with asthma/allergic disease in humans

5HTP blocks recruitment of eosinophils in four models of allergic inflammation and blocks increased airway hyperresponsiveness in models of allergic airway inflammation (18). Endothelial cells express enzymes for synthesis and degradation of serotonin (18, 102–106). There are two *TPH* genes for the metabolism of tryptophan to 5HTP which is then metabolized to serotonin and there are 14 receptors (HTRs) for serotonin (61, 62). To focus our studies on HTRs that may be involved in eosinophil recruitment during allergic diseases and regulation of airway function, we determined if there are *HTR*s or *TPH*s in human chromosomal locations that are also chromosomal locations associated with asthma/allergic disease in genome-wide association studies (GWAS). Interestingly, genes in the serotonin pathway (63, 107–109) are located within chromosome locations that are also associated with allergy/asthma (32–34, 36–38, 41, 42, 48, 49, 52, 55–57, 65, 66, 68–71, 110–132) (Table 1B), suggesting that this pathway may be an important pathway in regulation of asthma and allergic disease.

### HTR expression by endothelial cells and eosinophils

**Figure 1:**
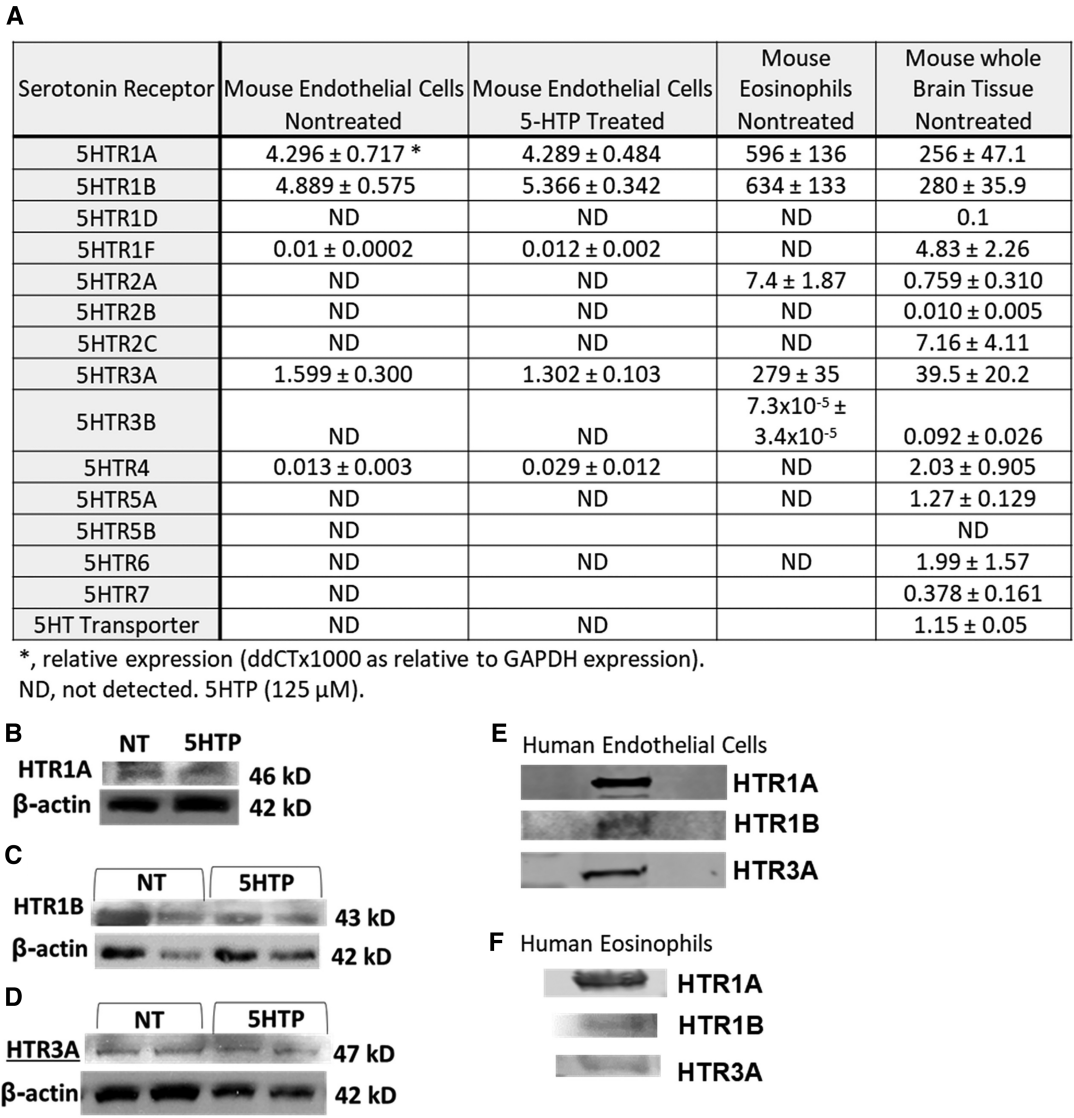
Expression of serotonin receptors in endothelial and eosinophils (mouse and brain control). (**A**) Mouse endothelial cell line, NJ1638 mouse eosinophils and C57BL/6 mouse brain tissue were analyzed for HTRs by qPCR. (**B–D**) Mouse endothelial cells with and without 5HTP (125 µM) were examined for HTR1A, HTR1B and HTR3A protein expression was analyzed by western blot. (**E**) Human microvascular endothelial cell and F) Human peripheral blood eosinophil protein expression of HTRs was analyzed by western blot. Panel (**E,F**) were not normalized to GAPDH because regulation of expression was not assessed.

We determined expression of serotonin receptors (HTRs) in endothelial cells and eosinophils by qPCR. Mouse brain tissue was a positive control for detection of HTRs. Mouse endothelial cells expressed HTR1A, HTR1B and HTR3A but not the other HTRs. Mouse eosinophils expressed HTR1A, HTR1B, HTR2A and HTR3A but not the other HTRs (Figure 1A). Also mouse endothelial cells were treated with 5HTP (125 µm) overnight as we previously described (18); this did not alter mRNA (Figure 1A) or protein expression of HTR1A, HTR1B or HTR3A (Figures 1B–D). These receptors are also expressed by human endothelial cells and human eosinophils in Figures 1E,F and as previously described (16, 31, 133–139).

### Pharmacologic inhibition of HTR1A and HTR1B blocked the 5HTP inhibition of eosinophil transendothelial migration

**Figure 2:**
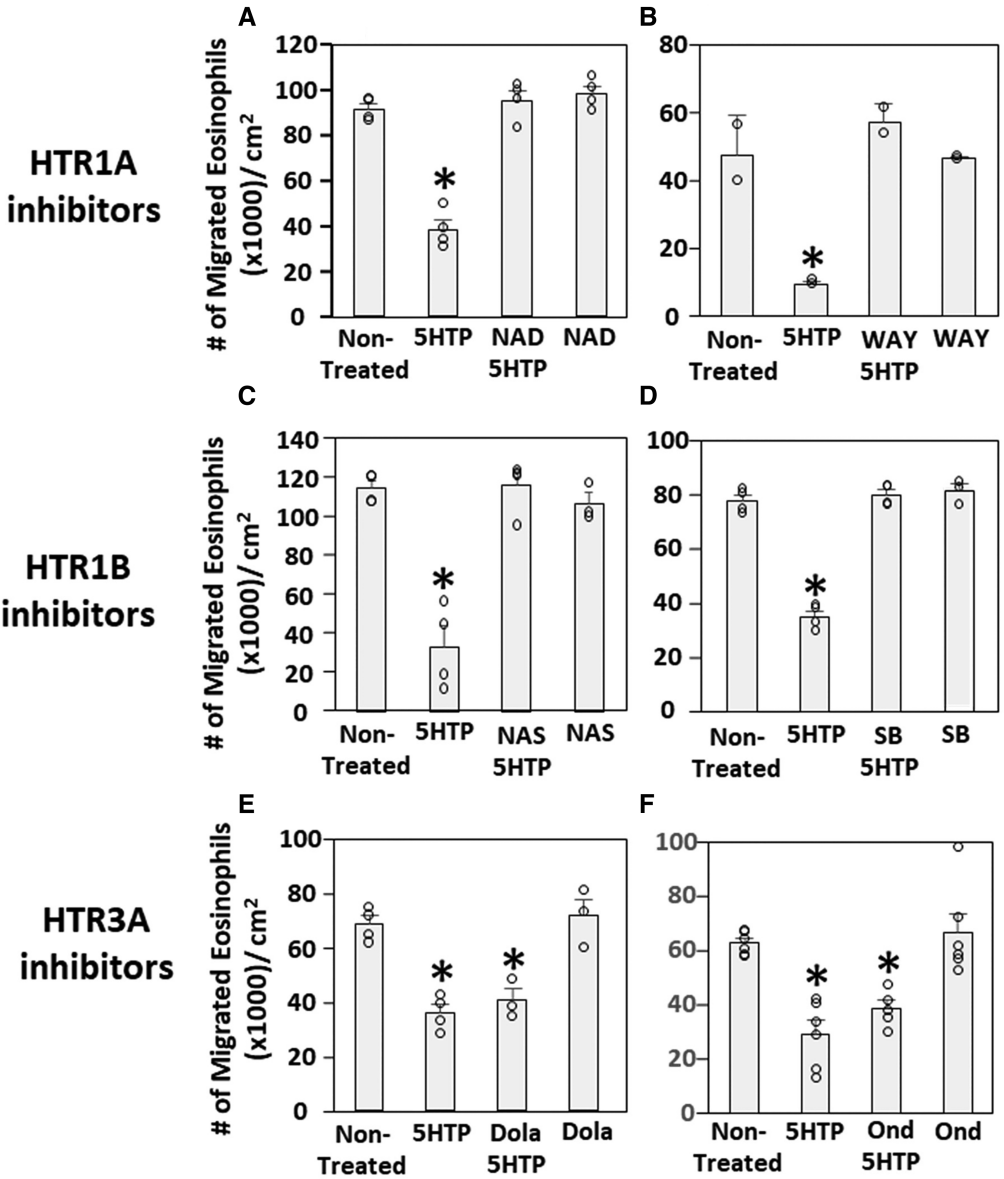
Pharmacologic inhibition of HTR1A and HTR1B blocked the 5HTP inhibition of eosinophil transendothelial migration. The pharmacological inhibitors were added to endothelial cells overnight and were present during the eosinophil transendothelial migration assay because these inhibitors are reversible. (**A,B**) HTR1A (6 nM NAD-299 and 150 nM WAY-100135) and (**C,D**) HTR1B (500 nM NAS-181 and 88 nM SB224289) blocked 5HTP (125 µM) inhibition of eosinophil transendothelial migration. In contrast, pharmacological inhibitors of (**E,F**) HTR3A (75 nM Dolasetron Mesylate and 62 nM Ondansetron Hydrochloride) did not block 5HTP (125 µM) inhibition of eosinophil transendothelial migration.

We have demonstrated that 5HTP is metabolized in endothelial cells to serotonin and that 5HTP overnight pretreatment of endothelial cells *in vitro* inhibits eosinophil transendothelial migration (18). In contrast, we demonstrated that 5HTP does not alter interaction of eosinophils with the endothelial cell apical surface *in vitro* (18) and does not alter expression of the adhesion molecules, including VCAM-1, or chemokines that promote eosinophil transendothelial migration (18), suggesting that 5HTP may regulate endothelial function during eosinophil transendothelial migration. In Figure 2, endothelial cells expressed HTRs. Therefore, we determined whether HTR1A, HTR1B and HTR3A regulate 5HTP inhibition of eosinophil transendothelial migration in a parallel plate flow chamber at flow rates in postcapillary venules, major sites of leukocyte recruitment during allergic inflammation. The endothelial cells were treated with highly selective pharmacological inhibitors of these HTRs at doses 10-fold the IC50 to sufficiently block receptors. The endothelial cells were pretreated with the HTR inhibitors for 15 min before addition of 5HTP and during the migration assay because the inhibitor binding to HTRs is reversible. Pharmacological inhibitors included the HTR1A inhibitors NAD-299 (NAD) and WAY-100135 (WAY), the HTR1B inhibitors NAS-181 (NAS) and SB224289 (SB), and the HTR3A inhibitors Dolasetron Mesylate (Dola) and Ondansetron Hydrochloride (Ond). The pharmacologic inhibition of HTR1A, HTR1B but not HTR3A blocked 5HTP inhibition of eosinophil transendothelial migration (Figure 2), indicating that HTR1A and HTR1B in endothelial cells or eosinophils mediated inhibition of eosinophil transendothelial migration.

### siRNA knock down of HTR1A and HTR1B expression in endothelial cells but not eosinophils blocked 5HTP inhibition of eosinophil transendothelial migration

**Figure 3:**
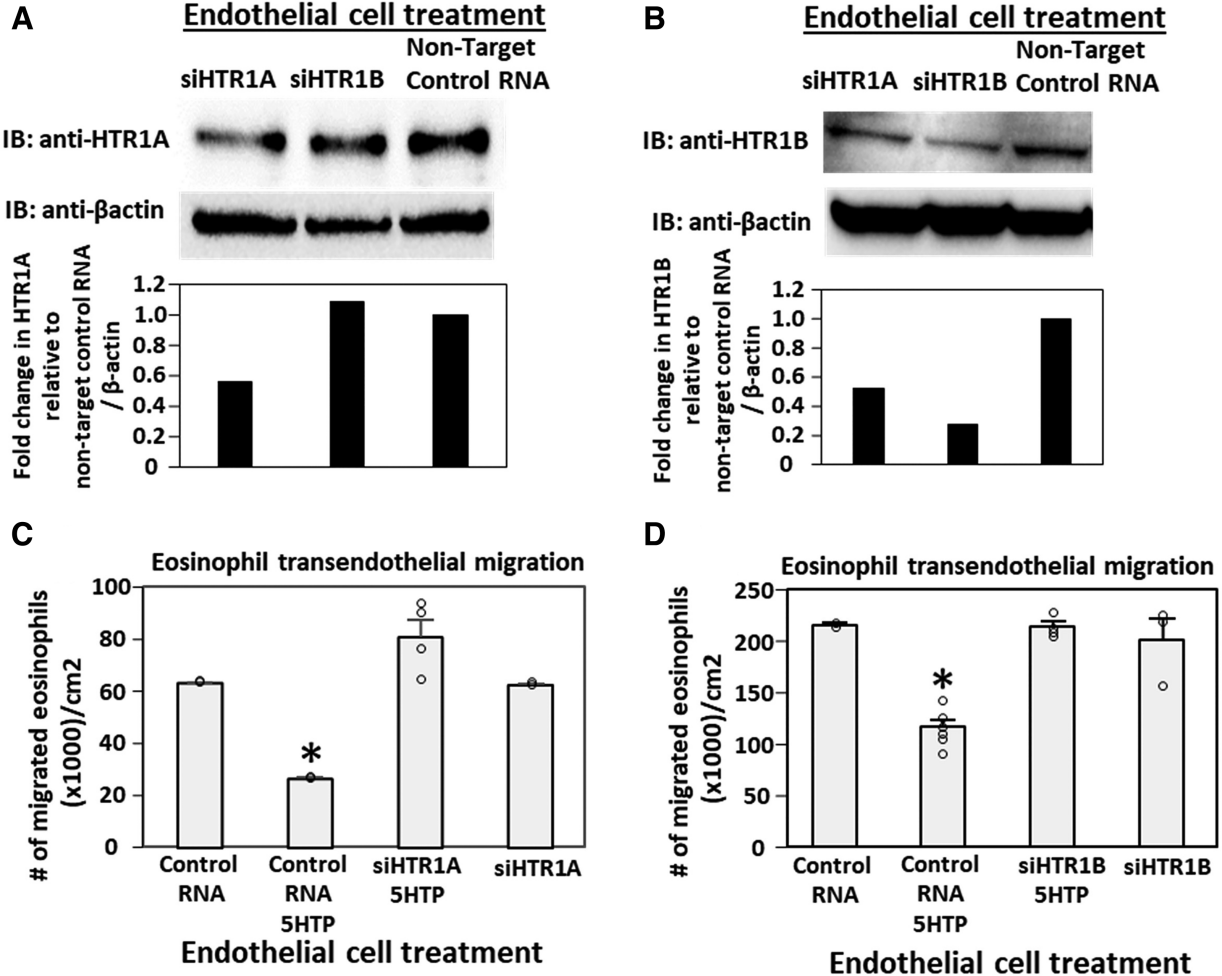
Endothelial cell HTR1A and HTR1B mediate 5HTP inhibition of eosinophil transendothelial migration. (**A,B**) HTR1A siRNA or HTR1B siRNA in endothelial cells knocked down HTR1A expression in the endothelial cells by western blot (HTR1A, 46kD; HTR1B, 43kD; βactin, 42kD) as compared to non-target control siRNA. Shown are representative blots of 2 experiments. (**C,D**) siRNA of HTR1A or HTR1B in endothelial cells blocked the 5HTP (125 µM) inhibition of eosinophil transendothelial migration. *N* = 3 for representative experiment of 2 experiments. *p < 0.05 compared to control siRNA.

Pharmacologic inhibition of HTR1A and HTR1B blocked 5HTP inhibition of eosinophil transendothelial migration in Figure 2. However, because the reversible inhibitors were present during eosinophil transendothelial migration, it is not known whether the HTRs in eosinophils or endothelial cells mediated the 5HTP inhibition of migration. To address HTR1A and HTR1B function in endothelial cells, the expression of these HTRs was knocked down in endothelial cells or in eosinophils by siRNAs. Also, because HTR1A dimerizes and HTR1B dimerizes (140), we determined expression of HTR1A and HTR1B for each siRNA knockdown. The siRNA for HTR1A only reduced the expression of the HTR1A (Figure 3A), but the siRNA for HTR1B reduced expression of both HTR1A and HTR1B (Figure 3B). Importantly, the knockdown of HTR1A or HTR1B in endothelial cells blocked 5HTP inhibition of eosinophil transendothelial migration (Figures 3C,D), suggesting that these endothelial cell HTRs functioned in 5HTP inhibition of transendothelial migration.

**Figure 4:**
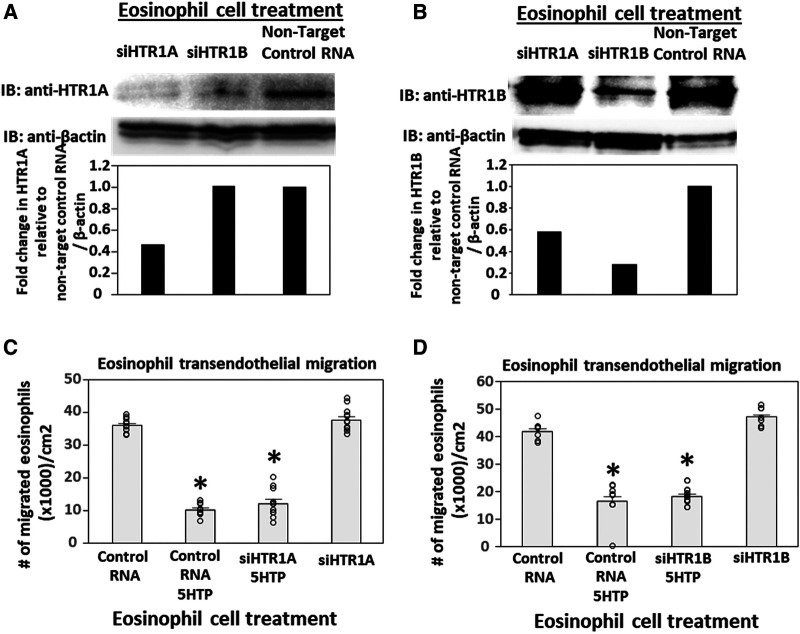
Eosinophil HTR1A and HTR1B do not mediate the inhibitory function of 5HTP on eosinophil transendothelial migration. (**A,B**) HTR1A siRNA or HTR1B siRNA in eosinophils knocked down HTR expression in the eosinophils by western blot as compared to non-target control siRNA. (**C,D**) siRNA of HTR1A or HTR1B in eosinophils did not block the 5HTP (125 µM) inhibition of eosinophil transendothelial migration.

To address the function of HTR1A and HTR1B in eosinophils, HTR1A siRNA or HTR1B siRNA was added to 5-day cultures of bone marrow generation of eosinophils because this is the stage of pre-eosinophils when IL5 was added to the culture. On day 8 of the culture, the cells were >85% eosinophils as determined by morphology and eosin staining of cytospins (data not shown). The treatment with siRNA for HTR1A and HTR1B during generation of eosinophils reduced expression of these HTRs in the eosinophils as determined by western blot (Figures 4A,B). Interestingly, siRNA of HTR1A or HTR1B in eosinophils did not block the 5HTP inhibition of eosinophil transendothelial migration (Figures 4C,D), indicating that HTR1A and HTR1B in eosinophils were not required for the 5HTP inhibition of eosinophil transendothelial migration.

### HTRs are expressed intracellularly in endothelial cells

**Figure 5:**
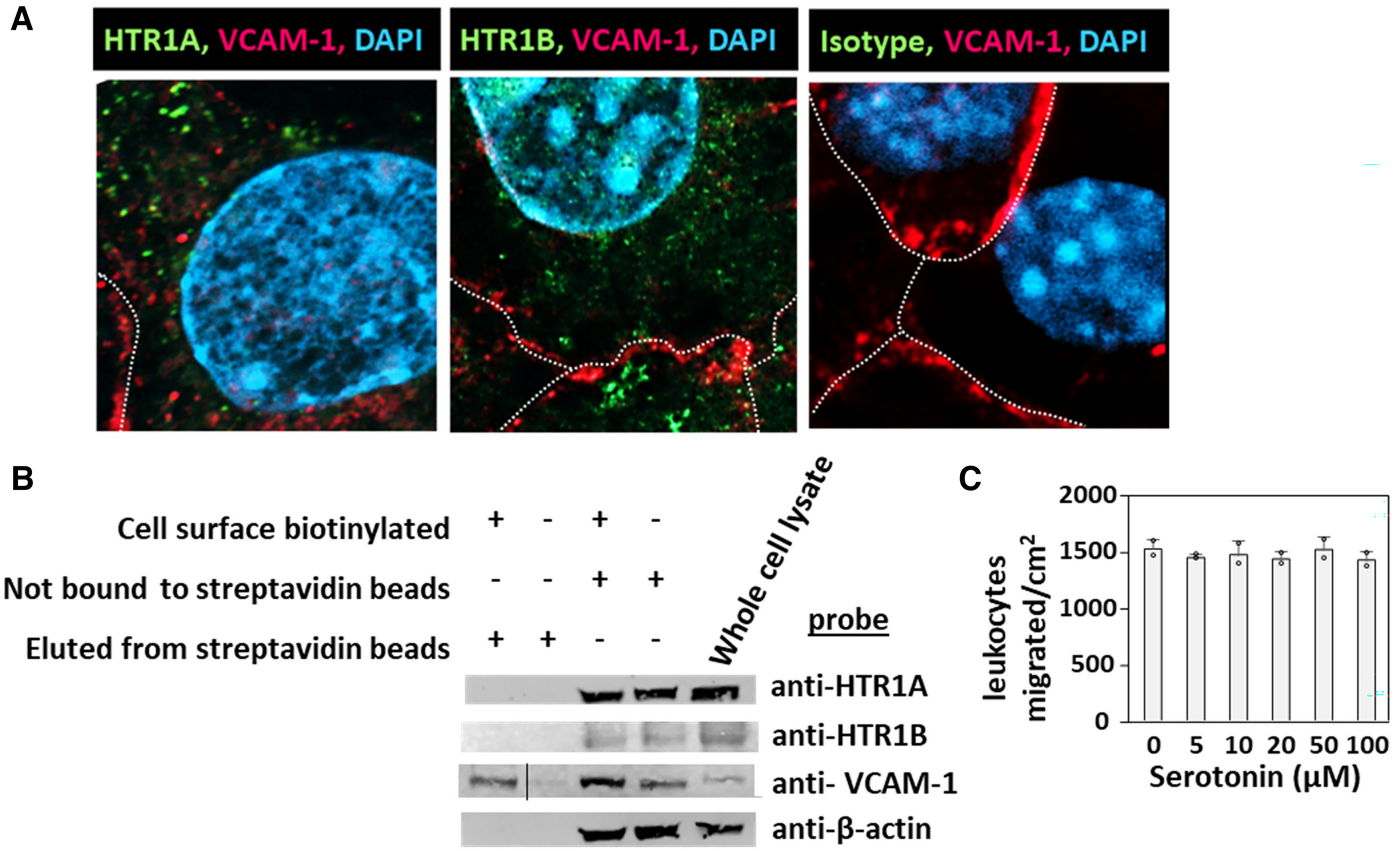
Exogenous serotonin did not block eosinophil transendothelial migration, consistent with intracellular HTR expression in endothelial cells. (**A**) Optical slices through the center of immunolabeled endothelial cell monolayers. Cells were fixed and immunolabeled with rabbit anti-mouse HTR1A, rabbit anti-mouse HTR1B or isotype control antibodies. Then the cells were washed and immunolabeled with FITC-conjugated goat anti-rabbit IgG. Then the cells were washed and immunolabeled with Alexa 647-conjugated rat anti-mouse VCAM-1 to mark the cell surface. The cells were cover-slipped with DAPI Prolong Gold to label the nuclei and coverslip the slides. The dotted white lines indicate location of cell membrane with VCAM-1 surface expression. (**B**) Biotinylation of endothelial surface proteins and western blot for HTR1A and HTR1B. (HTR1A, 46kD; HTR1B, 43kD; VCAM-1, 110kD; βactin, 42kD). The vertical line in the anti-VCAM-1 blot indicates removal of an empty lane where no sample was loaded on the gel. (**C**) Exogenous serotonin treatment overnight and during the transendothelial migration assay did not block leukocyte transendothelial migration.

HTR1A and HTR1B protein expression by mouse endothelial cells and eosinophils was determined by immunolabeling and confocal microscopy. Unexpectedly, these receptors, which have 7 membrane spanning domains, were not expressed on the cell surface but were expressed intracellularly with punctate localization in endothelial cells consistent with intracellular membranes (Figure 5A). To further demonstrate that the receptors were not expressed on the cell surface or expressed at low undetectable levels on the surface of endothelial cells, we biotinylated the endothelial cell surface, precipitated with streptavidin beads and examined expression of HTRs by western blot. The streptavidin-isolated biotinylated-proteins from the cell surface did not contain HTR1A and HTR1B by western blot, but the proteins from the cell lysate that were not pulled down with the streptavidin beads did contain these HTRs (Figure 5B), suggesting that the HTRs are not on the cell surface.

Because serotonin is transported across membranes by SERT but peripheral venule endothelium, which mediates leukocyte recruitment into inflammatory sites and synthesizes serotonin (102–106), does not express functional SERT (141) and because Figures 5A,B demonstrate intracellular localization of HTR1A and HTR1B, we determined whether exogenous serotonin regulated leukocyte transendothelial migration. We used a dose curve for exogenous addition of serotonin to the leukocyte transendothelial migration assay, because 5HTP, which is metabolized within these endothelial cells to serotonin (18), blocks both eosinophil (Figure 2) (18) and lymphocyte transendothelial migration (18) at 5HTP doses (75–125 µM). These doses are reported to regulate other functions of endothelial cells and other cell types *in vitro* (142, 143). Also, serotonin levels are increased up to µM levels by platelet activation during inflammation (31) and µM levels induce bronchoconstriction (83, 133, 144). To examine exogenous serotonin regulation of endothelial function during leukocyte migration, endothelial cells were treated overnight and during the transendothelial migration assay with serotonin (5–100 µM). Interestingly, serotonin, which does not cross peripheral venule endothelial cell membranes (141), did not block eosinophil transendothelial migration (Figure 5C), suggesting that metabolism of 5HTP to serotonin within endothelial cells functions within endothelial cells to block eosinophil recruitment.

### SNPs in *HTR*s and *TPH* associate with lower lung function in asthmatics

**Figure 6:**
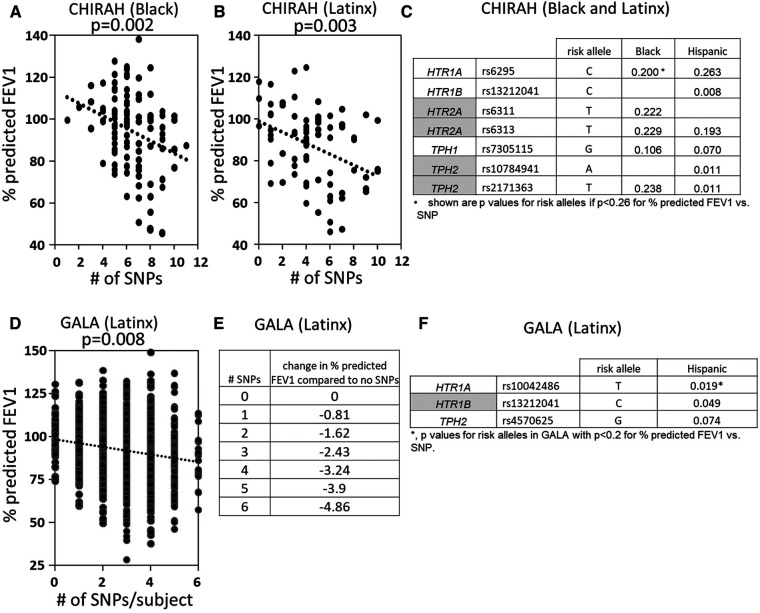
SNPs in *HTR*s and *TPH* significantly associated with lower lung function in humans. A polygenic index was calculated as the sum of SNPs with coding of 0, 1, or 2 for alleles with the SNP. SNPs included in the index had a nominal association with FEV1 (*p* < 0.2). (**A**) SNP index vs. % predicted FEV1 for CHIRAH black subjects. (**B**) SNP index vs. % predicted FEV1 for CHIRAH latinx subjects. (**C**) SNPs in *HTR*s and *TPH*s that had *p* < 0.26 for association with FEV1 for CHIRAH black and latinx subjects. (**D**) SNP index vs. % predicted FEV1 for GALA latinx subjects. (**E**) SNP index vs. change in % predicted FEV compared to no SNPs). (**F**) SNPs in *HTR*s and *TPH*s that had *p* < 0.2 for association of FEV1 for GALA latinx subjects.

5HTP is metabolized to serotonin in endothelial cells (18), 5HTP functioned through HTRs to regulate eosinophil transendothelial migration in Figures 3–5, 5HTP regulates lung function and allergic inflammation in the lungs in preclinical models (18), and in Table 1B, *HTR*s and *TPH*s are located in chromosome locations that are also found in GWAS studies of asthmatics. Moreover, some of these HTRs are adjacent to genes that regulate immune responses (Table 1B). Therefore, to determine whether the findings in the mechanistic preclinical studies on HTRs and lung function may extend to HTR function in asthmatics, we determined whether SNPs in *HTR*s and *TPH*s associate with lung function in asthmatics. We chose only SNPs in the *HTR*s that have functional consequences for HTR signaling or associate with behavior disorders (21–23, 26, 27, 29, 65–71) (Table 2). We chose a test asthma cohort CHIRAH from Chicago (85, 87) and a larger nationwide Latinx cohort, GALA (90), for proof-of-concept of the *HTR* and *TPH* genes with SNPs and associations with percent predicted FEV1. A polygenic risk score for subjects was generated using the SNPs in Figures 6C,F for CHIRAH and GALA. In CHIRAH, the polygenic risk score for *HTR*s and *TPH*s was significantly associated with lower lung function for adult black subjects (*n* = 103, 88 males, 15 females, age 31.8 ± 5.8) and adult Latinx subjects (*n* = 73, 54 male, 19 female, age 31.8 ± 5.4) (Figures 6A,B). These initial analyses in CHIRAH adjusted for duration of asthma in the Latinx subjects, and did not include adjustments other than ancestry in the African American analyses (due to the fact that smoking, inhaled steroid use, insurance status, severity classification, maternal education and household income were not confounders). In the larger cohort of Latinx asthmatic children GALA (*n* = 2,126, 1,128 female, 998 male, age 13.1 ± 3.5), the polygenic risk scores for HTRs and TPH were also significantly associated with lower lung function (Figures 6D,E). The GALA analyses were corrected for ancestry, smoking, inhaled steroid use, household income, and maternal education as potential confounders of the association. Although in the GALA Latinx dataset only one SNP overlapped with the CHIRAH SNPs and the index only consisted of 3 SNPs in the GALA sample compared to the 7 SNPs in the CHIRAH sample, the SNPs in the GALA cohort are within the same genes of the serotonin pathway as in the CHIRAH cohort. Given that the polygenic risk scores are not identical due to differences in SNPs genotyped due to different genotyping platforms, ethnic specific differences, and age in the cohorts in these associations, this represents a proof of concept and not a true replication of association.

**Table 2:** SNPs in HTRs and TPHs that have associations with behavior disorders.

Gene	SNPs	Reference
*HTR1A*	rs6295	(65, 66)
*HTR1A*	rs10042486	(66)
*HTR1A*	rs1364043	(66)
*HTR1B*	rs13212041	(67)
*HTR2A*	rs6311	(65)
*HTR2A*	rs6313	(68)
*HTR2C*	rs3813929	(69)
*HTR2C*	rs518147	(69)
*HTR2C*	rs6318	(70)
*HTR3A*	rs1062613	(71)
*HTR3B*	rs1176744	(71)
*HTR3B*	rs3782025	(71)
*TPH1*	rs1800532	(26, 27)
*TPH1*	rs7130929	(29)
*TPH2*	rs10784941	(70)
*TPH2*	rs11178998	(21–23)
*TPH2*	rs2171363	(70)
*TPH2*	rs4570625	(23)
*TPH2*	rs7305115	(23)
*TPH2*	rs7954758	(21)

## Discussion

Endothelial cells have an active function in eosinophil recruitment during allergic inflammation (145, 146). This recruitment of eosinophils in preclinical models is blocked by 5HTP supplementation (1). 5HTP is metabolized to serotonin within endothelial cells (18) by TPH1 (147). Furthermore, we demonstrate that the serotonin receptors HTR1A, HTR1B and HTR3A are expressed by endothelial cells and eosinophils. Inhibitors of HTR3A did not affect eosinophil transendothelial migration. However, the knock-down of expression of HTR1A or HTR1B in endothelial cells blocked 5HTP inhibition of eosinophil transendothelial migration. Also, the pharmacological inhibitors of the HTR1A and HTR1B receptors blocked 5HTP inhibition of eosinophil transendothelial migration. In contrast, knockdown of HTR1A and HTR1B in eosinophils did not modify 5HTP inhibition of eosinophil transendothelial migration, indicating that 5HTP inhibition of eosinophil transendothelial migration is mediated through HTRs in endothelial cells but not eosinophils. We also demonstrated that expression of HTR1A and HTR1B was within the cytoplasmic compartments of endothelial cells. Consistent with intracellular HTR localization in endothelial cells, 5HTP, which is metabolized to serotonin within endothelial cells, blocked eosinophil transendothelial migration but extracellular addition of serotonin, which is not taken up by endothelial cells, did not alter eosinophil transendothelial migration. Together these results indicate that to regulate leukocyte recruitment, 5HTP is metabolized to serontonin within the cytoplasm of endothelial cells and that this stimulates HTRs located within cytoplasmic compartments of endothelial cells, whereas HTR1A and HTR1B in eosinophils do not mediate 5HTP inhibition of eosinophil transendothelial migration. Furthermore, *TPH*s and *HTR*s are in chromosome locations that are near GWAS locations for allergic inflammation and asthma. In studies to extend HTR analyses to humans with asthma, we generated a polygenic index with SNPs that regulate TPH or HTR function or have been reported to associate with behavior disorders that associated with lower lung function. In humans with asthma, lung function is lower in asthmatics when the subjects are polygenic for SNPs in HTRs that alter HTR function or are associated with behavior disorders. This finding would suggest that TPHs and HTRs may serve as one possible link between asthma phenotypes and behavior disorders.

Allergic asthma occurs in about 80% of children with asthma and about 60% of asthma overall (3–5) and those with asthma have an increased prevalence of anxiety or depression (6–11). GWAS analyses of allergy and asthma have identified multiple chromosomal locations that are consistently associated with allergy/asthma (32–34, 36–38, 41, 42, 48, 49, 52, 55–57, 65, 66, 68–71, 110–132). These loci in Table 1B are associated with lung function and mediators of allergic inflammation, including IgE, eosinophils, IL4 and IL13. Interestingly, 10 of the genes for *HTR*s and *TPH*s (63, 107–109) are located near the dozen chromosome locations consistently associated with allergy/asthma (32–34, 36–38, 41, 42, 48, 49, 52, 55–57, 65, 66, 68–71, 110–132). This raises the question of whether the 5HTP/serotonin pathway is an important pathway in allergic asthma, and whether the perturbations of this pathway in subjects with allergic asthma may be a potential link explaining the increased rates of anxiety and depression observed in subjects with allergic asthma.

Anxiety/depression is often treated, with selective serotonin reuptake inhibitors (SSRIs) which are pharmacological inhibitors to block neuronal SERT transporter-mediated reuptake/removal of serotonin. It is reported that SSRIs inhibit eotaxin-induced eosinophil transendothelial migration through human dermal or lung microvascular endothelial cells *in vitro* (148), but the mechanisms are not known, especially since microvascular endothelial cells do not express a functional SERT (141). In a study of adult participants with both asthma and major depressive disorder, an SSRI had minimal effect on asthma control questionnaire scores and, for a subset of severe asthmatics, significantly lowered percent predicted FEV1 (149). Whether this is related to SSRIs having vasoconstriction side effects (75, 150) is not known. Also, in a mouse model of allergic asthma, serotonin derived from mast cells increases airway hyperresponsiveness (151). In contrast, 5HTP reduces airway hyperresponsiveness in several mouse models of allergic asthma (18), is not vasoconstricting (79–82, 152, 153), and still clinically effective for behavior disorders (154–164), but whether 5HTP can affect asthma is not known. Clinical studies with inhibitors of individual HTRs such as HTR1A or HTR2A have had only a small benefit for asthma (83), but this may reflect involvement of multiple HTRs during asthma. Because endothelial HTRs regulate inflammation which impacts lung function and because HTRs on smooth muscle cells regulate airway constriction and lung function, SNPs in *HTR*s and *TPH*s that are expressed in multiple cell types in the lung may impact lung function in asthmatics. We demonstrated that increased numbers of SNPs in *HTR*s and *TPH*s, which affect protein function or associate with behavior disorders, associated with lower FEV1 in asthmatic children and adults.

It is also reported that dietary supplementation with 5HTP blocks allergic inflammation in four mouse models, whereby the 5HTP was administered before or after sensitization for 2 different allergens or blocks eosinophil migration into the lungs after intratracheal IL4 administration (18). *In vitro* pretreatment of endothelial cells with 5HTP blocks eosinophil transendothelial migration (18); also 5HTP is taken up by endothelial cells and metabolized to serotonin within endothelial cells (18), but it was not reported whether endothelial cell generation of serotonin stimulates endothelial cell HTRs. We demonstrate that eosinophils and endothelial cells express intracellular HTR1A and HTR1B and that pharmacological inhibitors of these receptors block eosinophil transendothelial migration *in vitro*. We have previously reported that this eosinophil migration on the endothelial cells used in our studies is dependent on binding to VCAM-1, which reflects the VCAM-1 dependent recruitment of eosinophils into the lung *in vivo* (18, 145). The 5HTP inhibition of eosinophil transendothelial migration was through HTRs in endothelial cells but not eosinophils, because knockdown of HTR1A or HTR1B in endothelial cells blocked 5HTP inhibition of eosinophil transendothelial migration but knockdown of HTR1A or HTR1B in eosinophils did not block 5HTP inhibition of eosinophil transendothelial migration. Knockdown of either HTR1A or HTR1B in endothelial cells blocked the effects of 5HTP, which likely reflect that these HTRs can dimerize (140).

Others have reported that human eosinophils express HTR1A, HTR1B and HTR2A by RT-PCR (139). It is reported that treatment of human or mouse eosinophils *in vitro* with exogenous serotonin or an HTR2A agonist increases eosinophil chemotaxis and increases eosinophil rolling on recombinant VCAM-1-coated plastic surfaces (16, 139). Another report indicates that *in vivo* infusion of serotonin in mice, enhances eosinophil interaction with endothelial surfaces with increases in numbers of eosinophils rolling on the endothelial surface, decreases in eosinophil rolling velocity and increases in number of eosinophils adhered to the endothelium (16, 139), but transendothelial migration was not reported. We showed that exogenous serotonin did not alter eosinophil transendothelial migration, which is consistent with lack of functional SERT by venule endothelium (141) and our demonstration in this current report that endothelial HTRs are intracellular. It is reported that pharmacological inhibition of HTR2A in mice reduces eosinophil recruitment to lungs of mice challenged with the allergen chicken egg ovalbumin (OVA), but it was not shown whether plasma or tissue serotonin levels were increased in the mice. It another report, mice challenged with inhalation of OVA or the allergen house dust mite did not change serotonin levels in the mouse plasma, lungs, intestine or brain (18). In studies with 5HTP, supplementation of 5HTP *in vivo* does not increase plasma serotonin concentrations but decreases eosinophil recruitment into the lung during allergic inflammation (18); *in vitro* 5HTP is metabolized within endothelial cells to serotonin and decreases eosinophil transendothelial migration but does not alter chemokine-induced leukocyte chemotaxis or chemokinesis (18). We demonstrated that 5HTP inhibition of eosinophil transendothelial migration requires intracellular HTRs in endothelial cells and that in humans, SNPs in these receptors associate with lower FEV1 in asthmatic adults and children.

In conclusion, we have demonstrated on a cellular and molecular level that HTRs are expressed intracellularly in endothelial cells and that these receptors mediate 5HTP inhibition of eosinophil transendothelial migration (Graphical Abstract). Moreover, in asthmatic adults and children, a polygenic index of SNPs in *TPH*s and *HTR*s correlated with lower FEV1. A limitation is that the analyses in the Latinx subjects in CHIRAH was not corrected for ancestry due to limited genotyping being carried out in these subjects. Notably, similar polygenic associations were noted in the analyses of African American subjects in CHIRAH and the analyses in GALA II which were corrected for ancestry. Furthermore, the two human cohorts are not replication cohorts for the polygenic index because of differences in the population demographics and historical differences in genotyping platforms used. Nevertheless, both cohorts indicate that increasing the number of SNPs in the serotonin pathway associate with lower lung function within subjects with asthma. These studies form a framework for future studies to assess effectiveness of 5HTP for asthmatic subjects, especially since 5HTP has minimal side effects in other human studies (162), is not vasoconstricting (79–82, 152, 153), and is clinically effective in studies of anxiety and depression (154–160).

## Data Availability

The datasets presented in this study can be found in online repositories. The names of the repository/repositories and accession number(s) can be found below: https://www.ncbi.nlm.nih.gov/gap/, phs001274.v2.p1.
